# Giant Borderline Phyllodes Tumor Fungating Through the Skin as Fleshy Polypoid Outgrowths

**DOI:** 10.7759/cureus.61020

**Published:** 2024-05-24

**Authors:** Mitsuaki Yoshida, Akihiro Shioya, Emi Morioka, Masafumi Inokuchi, Sohsuke Yamada

**Affiliations:** 1 Department of Pathology and Laboratory Medicine, Kanazawa Medical University, Uchinada, JPN; 2 Department of Breast and Endocrine Surgery, Breast Center, Kanazawa Medical University Hospital, Uchinada, JPN

**Keywords:** giant phyllodes tumor, phyllodes tumor, macroscopic findings, fungating, polypoid outgrowths, breast tumor

## Abstract

We present the case of a 52-year-old female with a giant phyllodes tumor (GPT), which was fungating through the skin that showed fleshy polypoid outgrowths. Histological analysis revealed stromal atypia, mitotic activity, and stromal overgrowth; however, the tumor border was well-defined, and malignant heterologous elements were not observed. Therefore, as some but not all malignant histological characteristics were present, we diagnosed the patient with borderline GPT. In cases of phyllodes tumor (PT) with the unique gross findings of fungation through the skin as fleshy polypoid outgrowths, caution is required for the subsequent course because even if the PT is graded as benign histologically, a malignant process can occur. Pathologists should note that the sampling of the collection site and the ambiguity of the histological grading of PT may affect the final diagnosis of GPT. It is also important to perform surgery with adequate preservation of the resected margins to control recurrence for patients with GPT.

## Introduction

Phyllodes tumors (PTs) account for 0.3%-1% of all primary breast tumors and 2.5% of all fibroepithelial neoplasms [1]. Within this group, giant phyllodes tumors (GPTs) are even rarer, comprising 20% of PTs that grow to ≥10 cm [2]. According to the World Health Organization’s (WHO) classification, PTs are “tumors that fungate through the skin as fleshy polypoid outgrowths are invariably malignant.” However, without malignant heterologous elements, regardless of macroscopic findings, malignant PTs are diagnosed histologically if all the following features are present: marked stromal nuclear pleomorphism, stromal overgrowth, increased mitoses, increased stromal cellularity, and an infiltrative border. When some but not all adverse histological characteristics are observed, a diagnosis of borderline PT is made.

In this case, we encountered a GPT that was fungating through the skin that showed fleshy polypoid outgrowths. Pathological findings led us to ultimately settle on the diagnosis of “borderline.” However, differences occurred between such unique macroscopic and microscopic findings in the grading of malignancy. Herein, we discuss the pathological diagnostic issues of GPT.

## Case presentation

**Figure 1:**
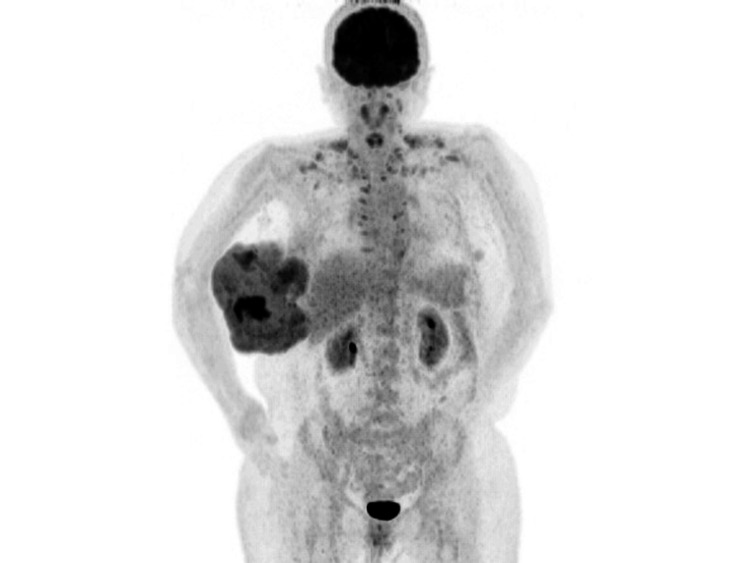
Positron emission tomography A giant mass was found in the right breast. Heterogeneous FDG accumulation was observed in the mass, and the maximum standardized uptake value was remarkably high at 12.89 in the area of strong accumulation FDG: fluorodeoxyglucose F 18

The patient was a 52-year-old female with no remarkable medical history who had developed a tumor in her right breast and underwent fine-needle aspiration cytology at another clinic seven years previously. Cytology results indicated that the mass was benign, and a fibroadenoma was presumed. She had not visited a medical provider since. However, after noticing a rapidly growing tumor on her right breast six months earlier, she visited the hospital with the chief complaint of the tumor breaking through the skin and exposing its components on the skin’s surface along with general malaise and anorexia. Clinical examination revealed a giant tumor in the right breast, which showed fleshy polypoid outgrowths fungating through the skin. Blood pressure was 123/104 mmHg, pulse was 127/minute, and temperature was 37.2°C. Blood tests showed a white blood cell count of 151,00/μL with a neutrophil percentage of 87.3% and a C-reactive protein level of 9.13 mg/dL. The clinical findings were considered inflammation associated with partial necrosis of the tumor site. The blood tumor markers were within reference values: carcinoembryonic antigen (CEA), 2.7 ng/mL; cancer antigen 15-3 (CA15-3), 7.3 U/mL; breast cancer antigen 225 (BCA-225), <20.0 U/mL; and National Cancer Center-stomach-439 (NCC-ST-439), <1.0 U/mL. A fluorodeoxyglucose F 18 (FDG)-positron emission tomography/computed tomography scan showed heterogeneous FDG accumulation inside the right breast mass, and the maximum standardized uptake value was remarkably high (12.89) in the area, showing strong accumulation (Figure 1). No metastases to other organs were observed.

A tumor biopsy revealed fibroepithelial lesions with a leaf-like growth pattern. Stromal cell density was heterogeneous and varied from low- to high-density areas with atypia. Based on clinical and biopsy findings, malignant PT was suspected. A total right mastectomy was performed. The weight of the right breast specimen was very high (2,905 g). The mass had held a space from the upper to the lower outer quadrants of the right breast and measured 26 × 21 × 13 cm.

**Figure 2:**
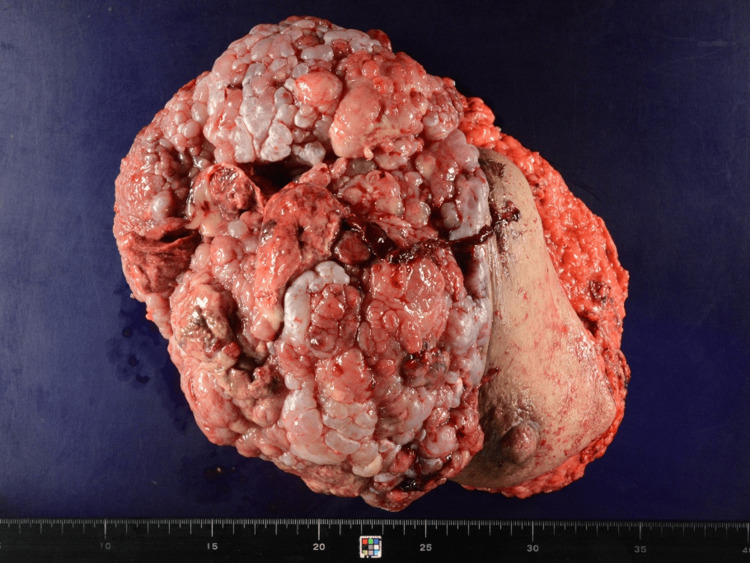
Right breast total mastectomy sample The mass was located in the upper outer and lower outer quadrant areas of the right breast and was 26 × 21 × 13 cm in size. Macroscopically, most of the tumor fungated through the skin as fleshy polypoid outgrowths. The central portion of the tumor showed hemorrhagic and necrotic changes

**Figure 3:**
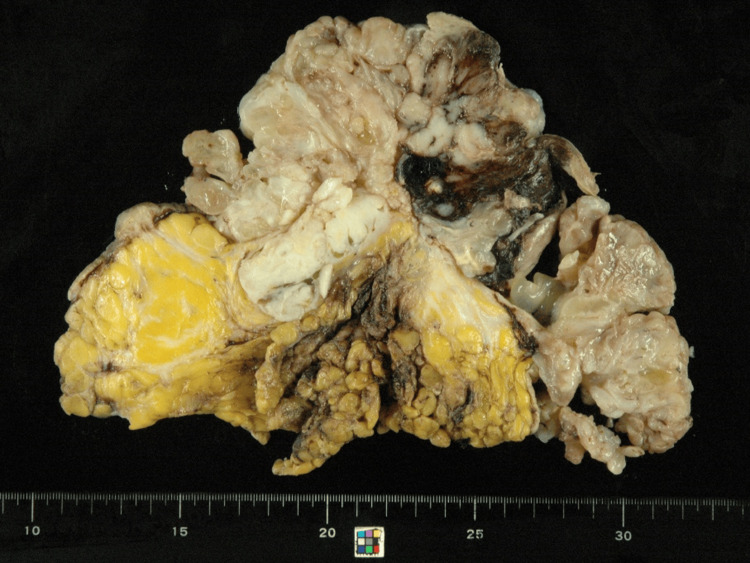
Macroscopic findings after formalin fixation Most of the tumor had a grayish-white, fibrous, elastic appearance with edematous changes. The areas with hemorrhagic changes were a mixture of hematoma areas showing a reddish-brown color and soft areas showing a grayish-white color

Macroscopically, most of the tumor had fungated through the skin as fleshy polypoid outgrowths. Its central portion of the tumor showed hemorrhagic and necrotic changes (Figure 2). The cut surface after formalin fixation revealed a grayish-white, fibrous, and elastic appearance with edematous changes. The areas with hemorrhagic changes were a mixture of hematomas showing a reddish-brown color and soft areas showing a grayish-white color (Figure 3).

**Figure 4:**
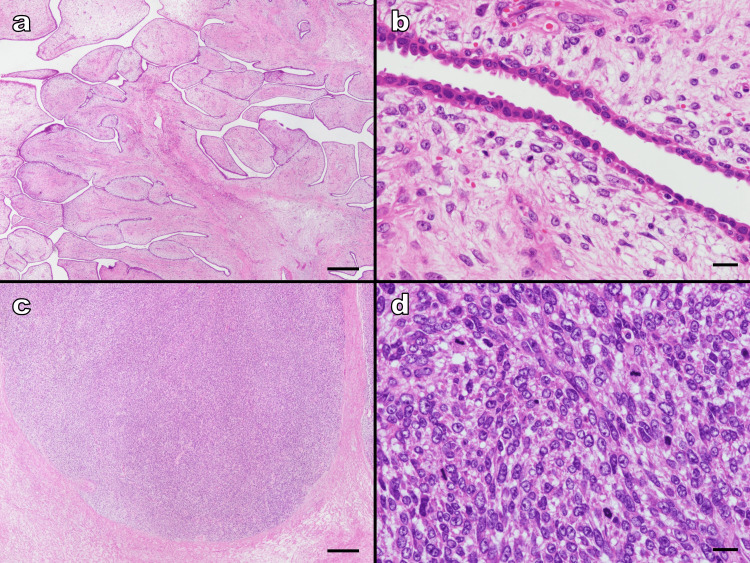
Hematoxylin-eosin-stained sample (a) In the fibrous parts, the tumor exhibited an exaggerated intracanalicular growth pattern with leaf-like projections extending into variably dilated elongated lumina (magnification, ×20; scale bar, 500 μm). (b) Stromal cellularity and atypical cells were scarce (magnification, ×400; scale bar, 20 μm). (c) No leaf-like structures were observed in the soft areas. Cell density in the stroma was elevated indicating stromal overgrowth. The border between the tumor and non-tumor areas was well-defined (magnification, ×20; scale bar, 500 μm). (d) Cells showing severe atypia with a high N/C ratio, distinct nucleoli, and atypical mitosis were observed (magnification, ×400; scale bar, 20 μm) N/C: nuclear/cytoplasmic

Histologically, the tumor exhibited an exaggerated intracanalicular growth pattern in the fibrous portion with leaf-like projections extending into the variably dilated and elongated lumina. Stromal cellularity and atypical cells were scarce (Figure 4a, 4b). No leaf-like structures were observed in the soft areas. The cell density in the stroma was elevated indicating stromal overgrowth (Figure 4c). Cells showing severe atypia with high nuclear/cytoplasmic (N/C) ratios, distinct nucleoli, and atypical mitosis were also observed (Figure 4d). Mitotic activity was observed as 46 mitoses per 10 high-power fields. No heterologous malignant elements were observed. The border between tumor and background mammary tissue was mostly PT component with mild atypia and well-defined. A few borders were in contact with components with severe atypia; however, their border appeared well-defined rather than permeative.

When applied to the histological features of the WHO classification of PT grading, stromal cellularity, stromal atypia, mitotic activity, and stromal overgrowth fulfilled the criteria suggestive of malignancy. However, the tumor border was well-defined, and malignant heterologous elements were not observed; therefore, the diagnosis of borderline GPT was made. After three years post mastectomy, the patient had not experienced recurrence.

## Discussion

We presented a case of GPT that fungated through the skin as fleshy polypoid outgrowths. Despite the WHO’s established classification of the clinical features of PT, no references in the literature have yet supported this finding [1]. Furthermore, skin ulcerations and fleshy polypoid outgrowths are not included in the histopathological malignancy grading. In several previous case reports, GPT cases with fungation through the skin as fleshy polypoid outgrowths have been graded benign regardless of their macroscopic findings and have not recurred [3-5]. Thus, we did not consider the skin findings in the histological classification of our GPT case, and there had been no recurrence after grading as borderline. However, there is one case of a GPT with fungating proliferation with histological grading of benign in which malignant pleural effusion has been reported, in which the patient died during the postmastectomy follow-up period [6]. Because we cannot exclude the possibility that GPT that fungated through the skin as fleshy polypoid outgrowths may have a malignant course, we suggest that careful follow-up is necessary for such GPT.

Some reports have shown larger PT size to be significantly correlated with recurrence [7,8], while a study by Yom et al. showed smaller size to be correlated with recurrence [9]. In a systematic review and meta-analysis by Lu et al., tumor size was not a significant risk factor for local recurrence [10]. Therefore, it remains unclear whether GPT is a risk for local recurrence compared to PT of less than 10 cm. The risk factors for the local recurrence of PT are more important for histological grade and margin status at excision than for size, and the risk factor for distant metastasis is malignant histological grading [1,2,10-12]. While histological grading is thus an important indicator for predicting local recurrence and distant metastasis, it must be taken into account that for tumors of large size such as GPT, pathological diagnosis can be affected by the site of sampling. These tumors are notoriously heterogeneous, and the adequate sampling of at least one block per centimeter of maximum tumor dimension with additional sampling of grossly heterogeneous areas is recommended [13,14]. In this case, 50 sections were sampled, with four of the five malignant histological features from the WHO classification being suggestive of malignancy. However, as the tumor border was well-defined, our PT grading remained “borderline” as per the WHO classification. However, it is difficult to prove that there is absolutely no permeative tumor border anywhere in GPT owing to the impracticality of preparing and microscopically searching entire tumors in cases of GPT.

In a survey from Tan et al., nearly half (49%) of pathologists did not require all the histological parameters for PT grading to be on the malignant end of the spectrum (as recommended by the WHO) before diagnosing malignant PT [13]. Although the guidelines related to PT diagnosis may appear straightforward, their application can be fraught with ambiguity [12]. Furthermore, how the subdivisions for each microscopic parameter interact to constitute the final grade is subjective [12,13]. In this case, we judged the tumor border to be well-defined as both areas with mild and severe atypia showed a clear border with the background tissue due to expansive growth. However, if another pathologist had reviewed this case, it may have been interpreted as a permeative tumor border condition in a severe atypical area or graded as malignant without assessing the finding of a tumor border as was done in the survey by Tan et al. [13] The histological grading of GPT should be done with an understanding of these histological diagnostic issues. Moreover, we consider it important to perform the surgery with well-preserved resection margins because local recurrence can be controlled regardless of the ambiguity of the histological grading of the GPT.

## Conclusions

This study presents a case of GPT that fungated through the skin as fleshy polypoid outgrowths. This unique clinical and macroscopic feature of PT required caution during the patient’s subsequent course. In addition, pathologists must understand that in the case of GPT, the sampling of the collection site and the ambiguity of the histological grading of the PT can affect the final diagnosis. It is also important to perform surgery with adequate preservation of the resection margins to control recurrence in GPT cases.
